# Is colonial heritage negative or not so much? Debating heritage discourses and selective interpretation of Kulangsu, China

**DOI:** 10.1186/s43238-022-00069-7

**Published:** 2022-09-23

**Authors:** Ran Wei, Fang Wang

**Affiliations:** 1 Department of Geography and Environment, London School of Economics and Political Science, Houghton Street, London, WC2A 2AE UK; 2 College of Architecture and Landscape Architecture, Peking University, No. 5 Yiheyuan Road, Haidian District, Beijing, 100871 People’s Republic of China; 3 NSFC-DFG Sino-German Cooperation Group on Urbanization and Locality (UAL); College of Architecture and Landscape Architecture, Peking University, Beijing, 100871 People’s Republic of China

**Keywords:** Heritage tourism, Colonial heritage, Authorised heritage discourse, Heritage interpretation, Postcolonialism, Critical heritage studies

## Abstract

Heritage is in essence dissonant, especially colonial heritage in postcolonial nations. Via questionnaire surveys and interviews, this study investigates Kulangsu in Xiamen, China, a colonial heritage site mainly developed in the 19th and 20th centuries, to unveil the local government’s authorised heritage discourse (AHD) of the site and how tourists perceive the colonial past of Kulangsu and construct their own heritage discourse(s). Results show that, when considering the colonial history of the site, neither the AHD promoted by the authorities nor the tourists’ lay discourses are necessarily negative. However, tension implicitly arises between the tourists’ demand for comprehensive heritage information and the authorities’ selective interpretation of the site. Although the AHD affects lay discourses to some extent, most tourists expect the authorities to present more complete and neutral information about heritage so they can reflect and forge their own conception of colonial legacies. From a critical heritage studies perspective, this tension reflects the power imbalance between the authorities and the tourists and reminds the authorities and heritage experts to rethink heritage tourism and conservation in terms of heritage interpretation. This paper, therefore, calls for additional reflection on the legitimacy of selective interpretation, which implicates a complex process of intricate reasoning that is underpinned by the power imbalance between the authorities and the tourists, ultimately resulting in an AHD.

## Introduction

In recent years, as colonial heritage sites have become popular tourism destinations around the world, the conservation and touristic development of colonial heritage have been increasingly situated in a postcolonial perspective through the lens of decolonisation (Jørgensen 2019). From this perspective, the tourism development of colonial heritage can be problematic. On the one hand, colonial heritage is associated with a colonial history that is usually deemed to be negative, traumatic, and humiliating for nations that were colonised (Youn and Uzzell 2016; Ifversen and Pozzi 2020). On the other hand, when people from countries that were once colonisers travel to previous colonies, this process is conceptualised as neo-colonialism that has significant impacts on local communities’ daily lives and shapes, or even determines, how heritage sites are conserved and developed, usually based on Eurocentric heritage conservation philosophy (Jørgensen 2019; Fortenberry 2021). Meanwhile, in places that were once colonised and are usually developing, the touristic development of colonial heritage sites has brought new opportunities in terms of economic growth and social advancement (Chambers and Buzinede 2015). In previously colonised nations, tourism-led neo-colonialism is sometimes countered through the use of colonial heritage as a resource for nation-building (Ifversen and Pozzi 2020).

These controversies depend on who *can and should* tell the stories of colonial heritage, make decisions about the conservation and touristic development of colonial heritage sites and benefit from it. These questions relate to the discourses used to address colonial heritage and the power relations embedded in its conservation and touristic development. The actors involved in the process do not necessarily come from the previously colonising nations and colonised nations, but they might well be within the previously colonised nations, if domestic tourism dominates international tourism (Jørgensen 2019).

The concept of authorised heritage discourse (AHD) is an insightful tool to scrutinise questions concerning heritage discourse and power relations, as it theorises the dominant heritage discourse of the authorities, heritage professionals, and elite class as ‘authorised’, which usually limits or suppresses subordinate groups’ expression of heritage value and use (Smith 2006). Bringing together postcolonial critiques and the AHD, this paper focuses on the interaction between tourism developers (the authorisers) and tourists (the subordinates) of colonial heritage sites. Using Kulangsu, a colonial heritage site and World Cultural Heritage Site located in Xiamen, Fujian Province, Southeast China, as a case study, this paper seeks to answer the following questions: (1) How do the tourism developers’ (the local government) AHD and the tourists’ discourses conflict with one another (or not)? (2) What are the roots of the conflicts and/or consensuses of these heritage discourses?

The objective and implications of this research address the relevance of examining the heritage discourses of different actors and their interactions in colonial heritage tourism. Through the discussion on these interactions, we can better understand the discrepancies that exist between tourists’ expectations of heritage tourism and what is provided to them by tourism developers. More importantly, we can critically shed light on the major debates in heritage conservation and tourism, including who can have a voice in these debates and how to interpret controversial heritage.

In Kulangsu, we find that the tourists’ heritage discourses are not necessarily in conflict with the AHD promoted by the local government and that colonial history is not always seen through a negative lens. However, most tourists expect the local government to present more complete and neutral information about the site to be able to ponder and develop their own ideas about colonial legacies. The findings echo previous studies in terms of acknowledging that tourists are not always passive recipients of the information provided by the authorities but may have a certain degree of independence in processing the information, even when the authorities’ AHD and the tourists’ heritage discourses are consistent (Smith 2006; Roppola et al. 2021). Furthermore, the findings indicate that the conflicts between the authorities and tourists are not rooted in colonial history per se but reflect the discrepancy between tourists’ demand for independent engagement with heritage and the authorities’ selective interpretation and presentation of heritage. Thus, it is worth reflecting upon the legitimacy (or illegitimacy) and impacts of the complex process by which selective interpretation arises.

## Theoretical framework

### Colonial heritage, postcolonialism, and heritage discourse

Postcolonial critiques in heritage tourism are relevant to this research in two ways. The first one is the problematic duality between the people from previously colonising nations and those of the previously colonised regions. Generally, postcolonialism in heritage tourism studies has mainly concentrated on the binary opposition between the First World and the Third World (Hall and Tucker 2004). The former represents the core, hegemony, and modernity, while the latter represents the periphery, resistance, and tradition. When people from the developed countries engage in tourism, they preclude the previously colonised people from constructing their own national identity, and as such, they have been criticised for engendering a perpetual ideology of colonialism (Park 2016). Some empirical studies have come from this vein to reflect on the neo-colonisation by tourists and developers from previously colonising nations in ways of underrepresenting the colonial past associated with the colonial heritage (e.g., Aggett and van de Leur 2020; Nelson 2020; Fortenberry 2021).

However, Jørgensen (2019) and Dang (2021) point out that the overemphasis on tourists from previous colonial powers may neglect the important impacts of domestic tourists. Indeed, the binary between the colonisers and the colonised is what Edward Said initially criticised in his ground-breaking book *Orientalism,* as the clear-cut difference between ‘Self’ and ‘Other’ is one of the very roots of colonisation in the first place (Said 1978). Such a dichotomy is therefore criticised as a misreading of postcolonialism (Wu 2020). Additionally, colonial heritage tourism can be much more complicated than tourists from previous colonial powers negatively affecting local communities’ identity construction. For instance, Cheer and Reeves’s (2015) case study in Fiji involves conflicts within local communities separated by ethnicities that are beyond the dichotomy of colonisers and colonised. Wolff’s (2021) research on colonial heritage in Serampore shows how local communities inscribed the colonial heritage into their local identities and used it as a way to express their discontent with today’s rapid urban change compared to the moderate development and ‘good governance’ of the colonial era, which is embodied in their colonial heritage. Zhang’s (2021) research in Harbin reveals a clear generational gap where the young generation embraces the colonial heritage of the city as the symbol of their culture more than the elderly do (for more on this topic, see Lu 2022; Chuva, Aguiar, and Fonseca 2022; etc.).

Jørgensen and Dang’s observation and critique are important in the context of China because when colonial heritage sites become tourist attractions there, they receive far more domestic than international tourists. The Chinese example is therefore able to contribute to postcolonial heritage tourism research by examining how domestic tourists and markets perceive colonial history and affect colonial heritage conservation.

The second way in which postcolonialism is relevant in this research is that postcolonial heritage studies has gradually taken into consideration the complex interplay between colonial heritage and memories by unpacking the ambiguities derived from heritage discourses and the associated conservation and management practices (Marschall 2008). This is where the concepts of heritage discourse and AHD can be raised. Heritage discourse rests on the premise that heritage is a discursive practice. What is categorised as heritage and what is not, and what values and meanings are represented by heritage are subject to people’s interpretation. The configuration of heritage discourse can include the oral and written interpretation, or more formally, provision of heritage concept, criteria, and value (Yu and Zhang 2020). It is through heritage discourse that the interpretation of the values and memories of colonial heritage is achieved.

The examination of heritage discourse implies that heritage is intrinsically dissonant (Tunbridge and Ashworth 1996). It is the power relations and value judgement of the actors within heritage practices that shape heritage discourse (S. Wang 2019). Therefore, conflictual heritage discourses may arise when there are power imbalances and diverse value judgements among the multiple actors. The dominant heritage discourse is captured by the concept of authorised heritage discourse (AHD), referring to a hegemonic heritage discourse ‘which is reliant on the power/knowledge claims of technical and aesthetic experts, and institutionalised in state cultural agencies and amenity societies…The “authorised heritage discourse” privileges monumentality and grand scale, innate artefact/site significance tied to time depth, scientific/aesthetic expert judgement, social consensus and national building’ (Smith 2006, 11). In other words, AHD is a product of the elite class that prioritises expert knowledge and the role of authorities, inevitably leading to other groups being muted, particularly underprivileged people.

From a postcolonial perspective, the attempt of postcolonial nations to rebuild their heritage discourse by highly selectively branding and propagating national heritage and memory landscapes is seen as a way to cope with cultural colonisation and imperialism and offset the impact of colonial heritage (e.g., Ifversen and Pozzi 2020; Zhou 2020). Tourists at colonial heritage sites are not always passive consumers of what is presented to them but may actively engage with colonial memories to form their heritage discourses (e.g., Park 2016; Lo 2018); they may also be relatively indifferent to the colonial past, receiving it instead as pure entertainment (e.g., Nelson, 2020). As David Lowenthal (1998) elaborated, heritage is different from history in the sense that history is what happened in the past, i.e., the historical facts, whereas heritage is what people say about the past, i.e., the interpretation of the facts. The interpretation, or the formulation of heritage discourse, is a subjective process that usually serves contemporary needs (Lowenthal 1985, 1998; Tunbridge and Ashworth 1996). In the process of transforming colonial history to colonial heritage during heritage tourism in postcolonial nations, the conflicts are more often caused by the way this transformation occurs rather than by the raw material, i.e., by the authorised heritage discourses of the authorities and the tourists’ ‘lay discourses’ (Parkinson, Scott, and Redmond 2016) rather than by historical facts from the colonial era (Dang 2021).

In summary, heritage discourse provides a lens through which to investigate the dissonance emerging from colonial heritage in postcolonial nations. This research seeks to expand the postcolonial critique of heritage tourism by focusing on domestic tourists and applying AHD as a theoretical framework to explore the contestation and negotiation of the authorised heritage discourse and lay discourses and the impacts of the interaction of those two heritage discourses during the development of colonial heritage tourism.

### Colonial heritage discourses in postcolonial China

After 1840, China remained a half-colonial and half-feudal nation for approximately a century; from this era, China has maintained a rich colonial heritage. Similar to some postcolonial nations that have struggled with addressing their traumatic and humiliating history of colonisation, China has undergone a big shift from the politics of excluding and destroying colonial heritage to embracing it as an important component in its heritage conservation system (H. Zhang 2018; S. Zhang 2018; Liu and Chen 2018). Such a shift has been shaped not only by the change in attitudes and methods of heritage conservation in China but also by ideological changes of the People’s Republic of China since 1949 (Zhou 2020).

It has been argued that, since Deng Xiaoping initiated the Reform and Opening in 1978, China’s economic system has combined market economy with socialism where diverse forms of ownership are allowed in addition to the mainstay public ownership. While younger generations who did not live through the revolutionary era have become relatively less familiar with Communism, Marxism-Leninism, and Mao’s socialist ideology compared to the earlier generations (e.g., Hung 2018; Svensson and Maags 2018). Following the opening up of the market, since the late 1980s and early 1990s, the Chinese Communist Party has gradually realised the appeal and importance of Chinese traditional culture and thus used culture to stimulate the sense of belonging and national identity among citizens (Oaks 2016).

The impact on colonial heritage is exemplified by the HSBC Bank Building in Shanghai that Liu and Chen (2018) used to illustrate how this colonial heritage was framed as the physical memory of China’s national humiliation in the 1950s and 1960s. As a result, the associated colonial history was eliminated in the AHD to facilitate a spatial reorganisation based on socialism and the development of a Chinese communist ideology. In the new era of holistic heritage conservation, the building has now been designated as a national heritage unit and has been preserved and rebranded as part of the sociocultural memory of the country.

Against the backdrop of the constant need for nation-building since 1949, some colonial heritage sites in China have become the ‘patriotic education base’ where government agencies, state-owned enterprises, and schools organise group tours to elicit nationalism and patriotism (Zhou 2020). Furthermore, at the onset, whether it was appropriate to exploit colonial heritage as an economic resource for tourism was controversial (Wang 2005). However, influenced by large-scale heritage commodification and guided by local governments’ pursuit of economic gains, many colonial heritage sites have ultimately and inevitably been used as tourist attractions (e.g., Chang 2017; H. Zhang  2018; Chauffert-Yvart et al. 2020).

Clearly, China’s national ideology has shifted to a more open-minded and cultural-focused way where Chinese traditional culture and history is playing an increasingly significant role in terms of boosting citizens’ cultural confidence and enhancing the nation’s soft power since the 1980s (Zhang 2010). Part of this shift is also the change in the AHD in the development of conservation and tourism policies for colonial heritage sites. Currently, the AHD of the Chinese central government regarding colonial heritage is to maintain a mutually beneficial relationship between China and other countries. Therefore, colonial heritage is promoted as the witness of cultural exchanges between China and other countries, such as the former Italian concession in Tianjin, which celebrates the flourishing of Italian culture in China (Chauffert-Yvart et al. 2020). In the meantime, the prevalence of consumerism offers a good opportunity for local governments to revitalise colonial heritage to fulfil tourists’ nostalgic feelings and imaginations towards the ‘mysterious’ and ‘colourful’ colonial era in the major cities of China (Ifversen and Pozzi 2020).

Regardless of what purposes colonial heritage tourism in China serve, the commonplace is that during tourism development, the value and history of colonial heritage are selectively (re)interpreted. Selective interpretation is omnipresent around the world with all types of heritage (Ashworth 1994). However, selective interpretation of colonial heritage is worth examining in particular as it is usually questioned whether the selective interpretation equals an embellishment of negative history. Furthermore, the ways in which selective interpretation and the embellishing of history may influence tourists’ experience and lay discourses as well as the conservation of colonial heritage is worth exploring (Tan and Choy 2020; Ernsten 2021). Wong’s (2013) research in Macau shows that because mainland Chinese tourists are not fond of colonial history, tour guides there usually omit colonial history during their colonial heritage tours. However, when they encounter tourists from other countries, colonial history is an important element of their interpretation. Wong, therefore, calls this phenomenon the ‘sanitisation of colonial history’ (Wong 2013, 915). In their research in Shameen, Guangzhou, Zhao and Zhang (2018) argue that background knowledge of colonial heritage may cause more negative feelings in tourists by lowering their joyful emotions and intensifying their anger, sorrow, and fear. However, knowledge of colonial history does not fundamentally change tourists’ objectives of seeking entertainment. In their research in Hong Kong, Wang, DiMeolo, and Du (2021) adopt a slightly different angle and contend that people’s heritage discourses regarding colonial heritage are influenced by various economic and political factors, such as the economic achievement of the government and society as a whole and diplomatic relations with various countries and regions.

These debates highlight that the AHD around colonial heritage in China is complex. The interaction between the AHD and tourists’ lay discourses are made even more complex as more contextual factors are usually involved (Zhang 2021). This research aims to contribute a new case study and new insight to untangle this complexity by looking at how the AHD and tourists’ lay discourses interact and how the interaction reflects deeper conflicts within the process of colonial heritage tourism development.

## Study site and methodology

### Kulangsu: a colonial heritage, tourist attraction, and World Cultural Heritage site

**Fig. 1:**
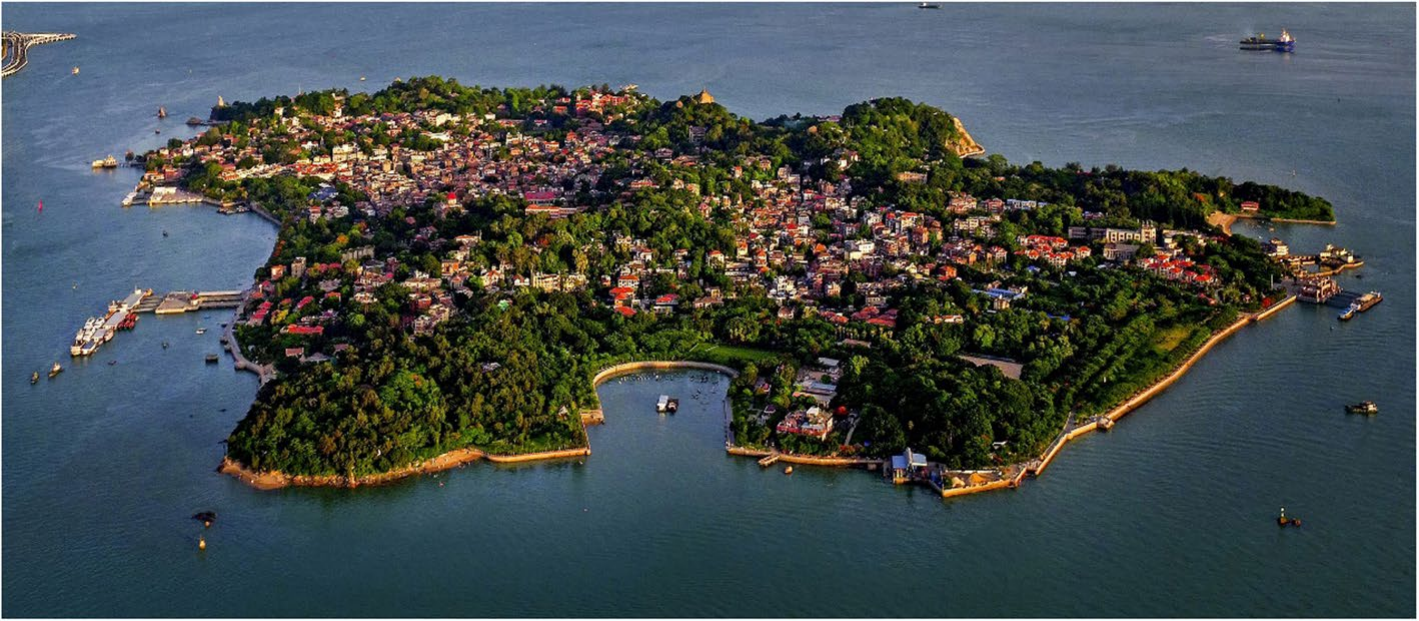
Bird's eye view of Kulangsu (Source: KULANGSU Administrative Committee. whc.unesco.org/en/documents/158147. Accessed 5 November 2021)

Kulangsu is a 1.87-square-kilometre island on the estuary of the Jiulong River (also known as Chiu-lung Chiang) in Xiamen, Fujian Province, Southeast China (Fig. 1).^1^ For this research, it is necessary to trace the colonial history of Kulangsu.

Since the 15th century, the island has been occupied by fishermen from Fujian Province. The climax of its development occurred from the 1840s to the 1940s. Defeated by the British army in the First Opium War in 1842, the Qing government was forced to open five port cities to international trade following the Nanjing Treaty, including Xiamen. Since then, the UK, the US, France, the Netherlands, Germany, Japan, etc. established embassies in Kulangsu and many foreign missionaries, businesspeople, and government officials came. These newcomers started by renting houses originally constructed by Chinese people and eventually built their own dwellings and public buildings. At the end of the 19th century, there were many embassies, churches, schools, hospitals, banks, and private dwellings of various architectural styles on the island, and from 1840 to the 1900s, Kulangsu gradually became an ‘international settlement’ where Chinese people and Western people lived spatially separated but in peace (W. Wang 2019).

**Fig. 2:**
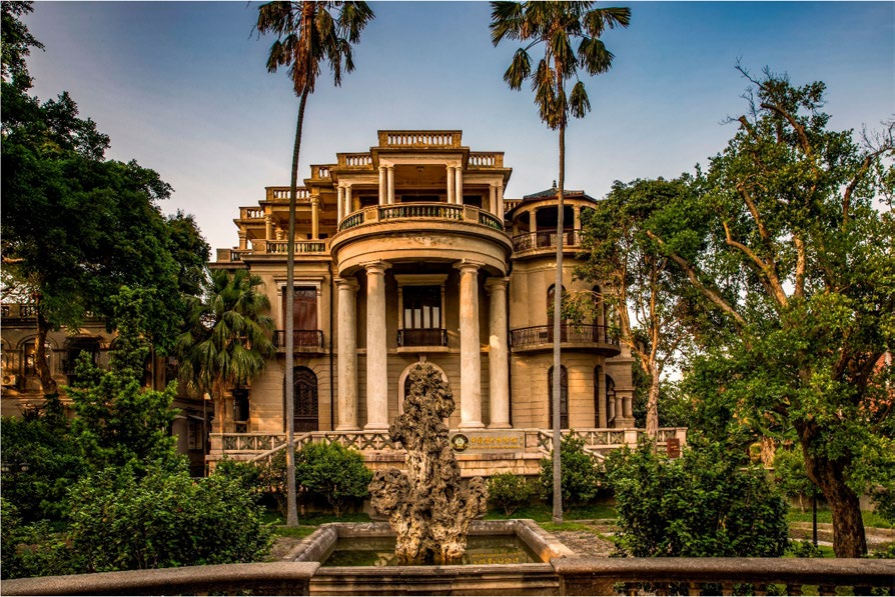
Amoy Deco Style building in Kulangsu (Source: Cultural Heritage Conservation Centre of THAD, photo by Qian Yi. whc.unesco.org/en/documents/158225. Accessed 5 November 2021.)

From the 1900s to the 1940s, because of the constant disputes between different colonisers about their rights in Kulangsu, the island became a ‘*gonggong dijie*’ (concession)^2^ that was comanaged by various countries. Different from other concessions in China at that time, Kulangsu’s sovereignty was still held by the Chinese government. This period saw many members of the Chinese diaspora come back to Kulangsu and invest in businesses in China. The Chinese diaspora gradually became the major force that physically transformed the island. During that time, the Amoy Deco architectural style, a mix of diverse architectural styles, was invented and flourished (Fig. 2; W. Wang 2019).

In addition to being a colonial heritage site, Kulangsu has also been a tourist attraction since the establishment of the People’s Republic of China in 1949. Since the 1980s, after more official planning and conservation work, Kulangsu was designated a National 5A Scenic Spot and saw a dramatic increase in touristic flows (W. Wang 2019). In 2008, the local government started to prepare for its World Heritage application. In 2017, Kulangsu was officially inscribed on the World Cultural Heritage list (Li and Chai 2018). It is estimated that more than eight million tourists have visited Kulangsu each year since 2010 (Lyu and Wei 2020).

Considering the development trajectory from the colonial era to the contemporary era, Kulangsu makes a good case study as a representative of the many colonial heritage sites in China that have transformed from places of past humiliation to popular tourist attractions. With the World Heritage designation, Kulangsu has entered the international stage, making its AHD a typical embodiment of China’s geopolitical agenda.

### Research methods

This research used literature research, questionnaires, and semi-structured interviews to collect data. First, academic papers, government reports, and planning documents were used to understand the historical background, conservation, and tourism development process of Kulangsu. Second, in July and August of 2021, 350 questionnaires were distributed to tourists, and 337 were effective. Thirty interviews were conducted with local planners, government officials, and tourists. The interviews lasted approximately 15 to 30 min. Each interview was recorded upon the interviewee’s consent. All participants were anonymised to protect their privacy. Due to the COVID-19 travel restriction in Xiamen in August 2021 when the fieldwork had been scheduled to occur, most of the questionnaire surveys and interviews were conducted online.

To summarise the heritage discourses of the two groups of actors, discourse analysis was conducted. All the interview transcripts were coded with NVivo to identify common themes and highlight keywords mentioned by different participants. These themes and keywords were then compared with those in the government reports and planning documents to trace the differences and similarities between the participants’ elaboration and the official records of Kulangsu. Questionnaires were used as supplementary data to measure the approximate proportion of each type of tourists.

## Heritage discourses of Kulangsu

### The AHD of Kulangsu

The AHD of Kulangsu is primarily determined by the local government, with an evolution between the times before and after the World Heritage application.

Government regulations and planning documents reflect that before the World Heritage application, the development and conservation of Kulangsu was focused on creating a well-known and desirable scenic attraction for tourists and on preserving the historic buildings on the island. These documents show that the conservation and development goals and measures were threefold. First, to control the population on the island, residents could only move out of Kulangsu with their household registration transferring elsewhere, while no new residents could transfer their household registration to Kulangsu. Second, to develop a more appealing scenic attraction for tourism development, industries that were irrelevant to tourism were not allowed to expand on the island or had to be removed from the island. Third, heritage conservation emphasised the restoration and maintenance of significant historic buildings, which were classified into different categories corresponding to different restoration measures (W. Wang 2019; personal communication). During this period, the AHD of Kulangsu was characterised as ‘emphasising scenic views while downplaying the community’ (*qiangjing ruocheng*), i.e., considering and using Kulangsu as a tourist attraction for tourism development with aesthetic value and economic potential rather than as a living community with various existing urban functions (Li 2015).

In the course of the World Heritage application, the AHD of Kulangsu shifted to *qiangcheng ruojing* (emphasising the living community while downplaying the scenic views). That is, the local government had come to recognise the significance of the living communities of Kulangsu along with their cultural practices and thus reoriented its conservation to focus towards both the tangible and intangible heritage, and both listed historic buildings and non-listed buildings. Meanwhile, tourism development has since been geared towards sustainable development to solve issues such as environmental degradation and pollution caused by uncontrolled tourism expansion (Li 2015; personal communication).

This change was partly motivated by the World Heritage application. While working on the application, local officials and experts gathered several times to decide on the Outstanding Universal Value (OUV) of Kulangsu. These meetings generated their new AHD of Kulangsu. However, this process was neither smooth nor straightforward. The first issue that arose was about Kulangsu’s name. Before being listed as a tentative World Cultural Heritage site, Kulangsu was known as Gulangyu, based on Chinese *pinyin*. Kulangsu was the name used by foreigners in the 1880s and has been more widely accepted by the international community. Therefore, Kulangsu was believed to better represent the ‘international discourse’, thus replacing Gulangyu as the official name for the World Heritage application (Li 2015).

Regarding the OUV of Kulangsu, when the island was initially nominated, the application dossier by the Chinese authorities claimed that Kulangsu complied with three of the criteria of the World Cultural Heritage listing: criterion (ii) to exhibit an important interchange of human values, over a span of time or within a cultural area of the world, on developments in architecture or technology, monumental arts, town-planning or landscape design; criterion (iii) to bear a unique or at least exceptional testimony to a cultural tradition or to a civilisation which is living or which has disappeared; and criterion (iv) to be an outstanding example of a type of building, architectural or technological ensemble or landscape which illustrates (a) significant stage(s) in human history.^3^ The ICOMOS review team acknowledged criterion (ii) quickly and criterion (iv) after receiving additional materials from China, and eventually rejected criterion (iii).

The way ICOMOS interpreted criterion (iv) regarding Kulangsu was different from China’s interpretation. Instead of Kulangsu constituting ‘an integrated and well-preserved historical island landscape, presenting distinctively the modernity that took the lead in those days and the modern habitat concept integrating Chinese and foreign cultures’,^4^ as elaborated by the Chinese team, ICOMOS believed that although based on China’s justification, Kulangsu ‘brings together a variety of typological approaches illustrating significant stages of history on Kulangsu Island’, but that ‘none of these landscape interpretations could be said of OUV’.^5^ ICOMOS’s acceptance of criterion (iv) was based on its own justification that Kulangsu is ‘the origin and best representation of the Amoy Deco Style’. Criterion (iii) was rejected on the grounds that Kulangsu’s historical development was far too specific and short-lived and thus inapt for representing China’s modernisation on a wider scale.^6^

Eventually, Kulangsu was inscribed under the name of ‘Kulangsu, a Historical International Settlement’ with an OUV based on criteria (ii) and (iv). The synthesis of Kulangsu’s OUV says:

Kulangsu is an exceptional example of the cultural fusion, which emerged from these exchanges, which remain legible in an organic urban fabric formed over decades constantly integrating more diverse cultural references. Most exceptional testimony of the fusion of various stylistic influences is a genuinely new architectural movement the Amoy Deco Style, which emerged from the island.^7^

From the official name and OUV of Kulangsu, it is clear that the current AHD of Kulangsu emphasises two aspects. The first is the value of mixed local communities that have historically been composed of different nationalities and ethnicities. The Chinese authorities are proud that Kulangsu is the first heritage site in the world to be listed under the banner of ‘international community’ and as a type of ‘community heritage’ (Li and Chai 2018). The second aspect is that the AHD appreciates and celebrates the historical fusion of cultures from different countries and regions. However, Kulangsu’s international community and cultural fusion were established by the colonisers during China’s half-colonial and half-feudal era, a period usually considered a time of ‘national humiliation’ in other contexts. This historical background has been omitted by the AHD of Kulangsu.

The process of formulating the AHD of Kulangsu, on the one hand, indicates that the Western-dominated heritage system has automatically imposed a dichotomy between the Global North and the Global South through differentiated heritage interpretation, which reflects the importance of the postcolonial critique that calls for the reconciliation between the West and the Other and the acceptance of more open and diverse heritage practices. On the other hand, can the omission of colonial history be considered historical cleansing? Zou and Xin (2017) contend that Kulangsu is a typical ‘shared built heritage’, defined by ICOMOS as ‘shared, or mutual, built evidence from around the world’.^8^ The appreciation and celebration of shared heritage should not be considered as neglect or beautification of traumatic past events but as an embrace of multiculturalism and interculturality and a move away from Western-centrism. The National Cultural Heritage Administration (previously known as the State Administration of Cultural Heritage) specifically held a ‘Kulangsu Shared Heritage Exhibition’ to illustrate the international value of Kulangsu. Overall, from the government side, Kulangsu’s AHD of emphasising cultural fusion while downplaying colonial occupation appears to be justified.

### Tourists’ lay discourses of Kulangsu

When examining the tourists’ heritage discourses of Kulangsu, this study finds that most tourists participating in this research paid more attention to the historic buildings, natural landscape, and traditional foods specific to Kulangsu and Fujian Province as the three major elements that constitute the heritage value of Kulangsu for them. When asked about colonial history and whether colonial history contributes to the heritage value of Kulangsu, approximately half of the questionnaire respondents indicated that they knew nothing or very little about Kulangsu’s colonial past. These tourists never truly considered whether colonial history was part of Kulangsu’s heritage value. However, the other half indicated that they knew more or less or very well about Kulangsu’s colonial past but not all of them believed colonial history to be relevant to Kulangsu being considered heritage. In some follow-up interviews, tourists expressed diverse opinions. For instance:

Is colonial history part of heritage value? I’m not sure. I always thought of heritage value as something good, you know, things that are visually beautiful and important and that make us happy about, oh and old (*TR03*).

Well, colonial history made Kulangsu what it is currently. If it weren’t for the foreigners, Kulangsu wouldn’t have these many buildings with different styles. Some of them look funny, but special, definitely. Of course, colonial history is important here. But I’m not saying that colonisation is good. It’s just that if you think of the heritage value of Kulangsu, the colonial past is unavoidable (*TR08*).

Tourists’ opinions about the colonial history and heritage value of Kulangsu lead to the next question of whether the AHD promoted by the authorities is seen as deliberate neglect and a sanitisation of history. Among the questionnaire respondents, only 18% strongly believe that the colonial history of Kulangsu is ‘bad’, ‘negative’, ‘humiliating’, and ‘traumatic’. One-third of them believed otherwise. Another one-third adopted a neutral position. And 13% indicated that they had never thought of this issue. Meanwhile, 27% criticised the authorities’ AHD for neglecting history and lacking objectivity and completeness. However, 43% accepted the AHD as a normal and acceptable interpretation of the colonial history of Kulangsu. The rest of them admitted that they had never given a thought to the issue or that it had nothing to do with them since it was a government’s decision. During the interviews, the participants also showed a relatively high level of acceptance of the AHD that had been promoted by the authorities in Kulangsu:

Frankly speaking, there is a certain degree of overlooking. But it’s normal and acceptable because it complies with the narrative of ‘World Cultural Heritage’ (*TR21*).

Well, different people clearly have different things to think about. Now we all have more and more channels to get all the information we want. I know that what the government says is just one side of the story. So, I wouldn’t say this it is purposely overlooking history. They just tell you what they want to tell, and that’s what we all do (*TR14*).

You know, it is not necessarily bad to not talk about colonial history, right? We have to maintain our relationship with other countries. We can’t just hold on to the past and hold a grudge all the time. We, as part of the world, need to move forward with others together. So, I understand (*TR08*).

Interestingly, the interviews and questionnaires revealed that when tourists agreed with Kulangsu’s AHD, it was based on the knowledge of colonial history they had acquired from other sources, for example through reading and searching on the internet and from speaking with residents of Kulangsu, rather than from what is officially presented on site by the authorities through pamphlets, signage, and exhibitions. Their acceptance was also based on their consensus that China needs to maintain a good relationship with the previous colonisers, rather than on whether colonial history is good or bad or whether colonial history is part of the heritage value of Kulangsu.

As for their own heritage discourses of Kulangsu, three types of tourists emerge: passive acceptors, active constructors, and pure fun-seekers. The passive acceptors are those who rarely think on their own but fully accept what is presented to them by the authorities:

Yes, we read the signs. I think they are quite informative. If you ask me how to understand Kulangsu as a heritage site, I will say that I’m with them. What they say is what I believe (*TR11*).

Active constructors, on the other hand, usually independently obtain various information and formulate their own understanding of the heritage:

I think the stories about some famous people are not very well told. I think these famous people and their background are also an important part of the value of Kulangsu. I would want to see more… Actually, what I know is from my own searching on the internet. The signs don’t say much… (*TR05*).

Fun-seekers are those who come and visit only to enjoy the natural landscape, taste the food, and take fancy photos for entertainment:

I don’t think it [Kulangsu] is a heritage site. Is it? I don’t know. I come here because it’s popular, and it’s beautiful, you know, as a scenic spot, a *wanghong di* to *daka* [Internet-famous site for punching in] … If it weren’t for you asking me all these questions, I would know nothing about the culture and history and stuff (*TR10*).

It is difficult to generalise one lay discourse of the tourists, as every tourist has his or her own experience, perception, and interpretation. What they did have in common is that most of the questionnaire respondents and interviewees wanted to see more about the colonial history of Kulangsu to think more about the culture and history of the island and formulate their own heritage discourses; this is true of even the passive acceptors and fun-seekers:

If they tell us more, maybe my opinion would be different, for sure. Now we don’t know much, so better not say stupid things. But I would love to see more, of course. You know, it’s our country’s history. We should know it (*passive acceptor, TR11*).

Now I feel guilty about not knowing anything about it. We learned all those colonial things in history class. We know the First Opium War, the Second [Opium War], the Eight-Power Allied Forces, and so on. I mean, come on, we are Chinese. Every Chinese knows this. Although I wasn’t a good student back in school [laugh]. But I really didn’t know that Kulangsu was part of that history. I’m surprised they [the AHD promoted by the authorities] didn’t say much. I would love to be educated on this matter (*fun-seeker, TR10*).

In summary, tourists’ lay discourses of Kulangsu are in agreement with the AHD in the sense that tourists do not necessarily consider its colonial history to be negative, and they do not necessarily receive the AHD as a sanitisation of history. Nevertheless, they do want more information regarding the colonial history of Kulangsu to independently think about Kulangsu as a heritage, particularly a colonial heritage.

## Consonance and dissonance in heritage discourses of Kulangsu

### Colonial history, AHD, and lay discourses

Comparing the tourists’ lay discourses of Kulangsu and the AHD, it is worth asking why it appears that they are generally not in conflict, especially regarding the colonial history of Kulangsu, which is not deemed to be so ‘negative’ by either the authorities or the tourists.

For the authorities, they do not fully control the construction of the AHD but are actively engaged in pursuing the international heritage discourse to have Kulangsu inscribed on the World Heritage list. In previous cases where the Chinese government attempted to have certain Chinese heritage sites listed as World Heritage, the team in charge of the application was believed to have taken a cynical attitude towards the World Heritage criteria. People on the team were well aware that they were ‘playing the game’ with international heritage experts following the Eurocentric rules set up by Western countries. There were certain aspects of heritage value derived from Chinese traditional culture that were not comprehensible by ICOMOS experts. Hence, they had to tailor the narratives to fit into the World Heritage framework (e.g., Zhang 2017; Yan 2021). Similar phenomena have been observed in other countries, especially countries in the Global South (Nugteren 2020). During the negotiation process between ICOMOS and the Chinese authorities about Kulangsu’s OUV, a similar situation arose, where the interpretation by ICOMOS overtook the initial interpretation made by the Chinese team and eventually constituted the Kulangsu’s AHD. Lyu et al. (2017) point out that the reason for China nominating Kulangsu as World Cultural Heritage is partly to acquire wider international recognition and enhance the cultural self-awareness and cultural confidence of Chinese citizens. Therefore, Kulangsu’s conservation and tourism development translates the geopolitical objectives of the nation, not merely the socioeconomic and cultural objectives. Considering China’s geopolitical position, it is perhaps not wise to make the colonial history of Kulangsu prominent in its AHD.

**Fig. 3:**
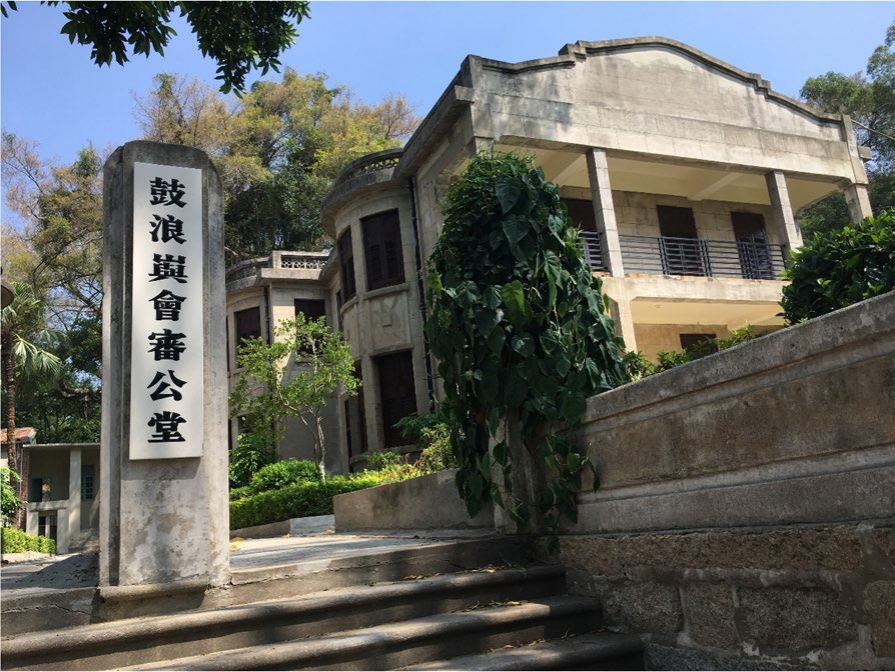
The former Kulangsu Mixed Court, 2018 (Source: the authors)

The backstage story of the World Heritage nomination and designation of Kulangsu is not well known by tourists. However, for the active constructors who do know about the colonial history of Kulangsu, China’s urgent need to maintain a peaceful and mutually beneficial relationship with other countries is well understood. This is one of the reasons why tourists are not bothered when the authorities downplay Kulangsu’s colonial history. Another reason is that compared to other ‘radical’ concessions in China, which were spatially and politically occupied by the colonisers and witnessed many bloodsheds during the colonial era, Kulangsu was a ‘moderate’ concession. As introduced by one tourist:

We had sovereignty in Kulangsu back then, you know. It’s a huge difference, although the colonisers might think otherwise. Actually, Kulangsu was indeed a place where foreigners and Chinese and members of the Chinese diaspora lived together without many evident conflicts. One exception might be the court [the Former Kulangsu Mixed Court, Fig. 3], you know. It was dominated by colonisers, so of course, the cases were solved without justice. The judges would rule in favour of the foreigners, not Chinese people, obviously (*TR23*).

The lay discourses of the passive acceptors and fun-seekers, who usually do not have enough knowledge about the colonial history of Kulangsu, are influenced by the AHD that is presented on the signage and in exhibitions on the island. When speaking of colonial history, what these tourists agree upon is that with the revitalisation of China in recent years, self-confidence has indeed been boosted by China’s achievements in all aspects. Feelings of hatred towards the colonisers become unnecessary:

I’m proud of our country’s accomplishments. Our traditional culture is marvellous. We don’t need to curse others to get powerful, to appear strong. We ARE strong (*TR02*).

Their assessment of colonial history also becomes more neutral as tourists are more educated:

When we were young, colonisers were all evil…But now, I think we need to rethink that history. I’m not defending colonisation. But there are bright sides. I mean, we were forced to open our doors and then started our fight and revitalisation. It was a chance to develop back then, and it is now a motive to remind us that we can’t go back to that time. We need to get stronger. This is the bright side (*TR08*).

Considering the necessary compromise that the Chinese authorities had to make for the World Heritage designation, the AHD chose to focus on cultural fusion and exchanges rather than on colonial disputes. In the face of the international geopolitical context and China’s domestic development achievements, tourists are comfortable re-examining the island’s colonial history with leniency and neutrality when formulating their lay discourses. Nowadays, both the authorities and the tourists find that Kulangsu’s colonial heritage is not that ‘negative’.

### Selective interpretation: from history to heritage

The consensus between AHD and lay discourses regarding the colonial history of Kulangsu does not mean that there is no conflict between the authorities and the tourists. The implicit contestation is centred on the selective interpretation of that colonial history. More specifically, tourists are dissatisfied with the authorities’ omission of colonial history in their tourism advertisements. For them, there is a difference between discussing colonial history with the international community and discussing colonial history with Chinese citizens:

Before I came here, I didn’t know about the colonial thing of Kulangsu. Only when you gave me the questionnaire and asked me the questions, I learned that Kulangsu has this sort of history…I think I get why I don’t see much about this history from the tourism promotion…We have foreign visitors here as well. Let’s earn their money by attracting them to tour China. But we are Chinese; we should know our history (*TR10*).

This contestation comes down to the long-lasting debates in critical heritage studies: whose heritage, who has the right to interpret it, and how it is interpreted (Waterton and Smith 2010). In the Kulangsu case, many tourists, even passive acceptors and fun-seekers, expressed their expectation of seeing more information about the colonial history of Kulangsu so that they can have their own understanding of the heritage instead of fully accepting what the authorities say:

Now since you ask, this pushes me to think. The reason I accept what is presented is that they don’t say much, and I don’t know that there is more. If they threw in some intriguing points, I would be motivated to learn more. How nice would that be! (*passive acceptor, TR12*)

Travel is for fun. I don’t want to be burdened by all the knowledge and history stuff. They are boring. We are here to taste the food and experience the culture and people’s lives here…But you know what? The history is part of the culture. It would be missing a chunk of the culture if the colonial history was not introduced to us. It feels like I watch a TV series, and never finish it… (*fun-seeker, TR09*).

The tourists’ responses imply two points. The first one is important for heritage tourism. While engaging in heritage tourism, some tourists have a strong desire to be independent in terms of learning about the heritage and interpreting it, especially those with more background knowledge about it and a high level of education. Heritage tourism is in essence different from other tourism types because heritage as a tourist attraction assumes more cultural connotations than entertainment functions. The second point is important for heritage conservation as a whole, not simply for heritage tourism development. That is, the interpreters of heritage and methods of interpretation are usually dissonant. The Kulangsu case echoes the theories, concepts, and arguments put forth by other heritage scholars mentioned in the theoretical framework section of this paper. The conflict between the authorities and the tourists about Kulangsu is not grounded in what happened during the colonial era, not in the ‘history’ per se. The conflict emerges out of the transformation process from ‘history’ to ‘heritage’. In heritage conservation and tourism, the authorities and heritage experts are usually the power-holders and the only voice, whereas the tourists are put in a passive position where they accept what is presented to them. Their independence is largely limited by the uneven power relations; hence, they are not able to turn ‘history’ into ‘heritage’ on their own and as they wish.

These two points push us, heritage experts and authorities, or ‘power-holders’, to think about how to use our power and whether it is possible to redistribute that power. On the one hand, although selective interpretation is inevitable in heritage conservation and tourism development, it is worth questioning what should be selected and what should be discarded. Is it fair that selective interpretation only takes into account the contemporary needs of the nation as a whole? Does this kind of selective interpretation risk damaging the cultural significance of heritage? On the other hand, who decides what to select and what to discard? To solve these issues, in practice, there could be more public consultations and participation programmes and initiatives to open the floor to a wider range of stakeholders, including tourists, residents, and business owners. In fact, to supplement the function of local government agencies and solve multiple social issues at the grassroots level, the local government and community associations co-established a Public Council that consists of residents, public agencies, and businesspeople to collectively contribute to the governance of Kulangsu (Wang 2017). This type of grassroots-level institution is a starting point for the inclusion of more actors and functions to facilitate more inclusive and diverse tourism management and heritage conservation.

## Conclusion

This paper combines postcolonial critiques concerning heritage studies and the authorised heritage discourse framework and uses Kulangsu as a case study to explore the interaction between the AHD of the authorities and the tourists’ lay discourses during heritage tourism. Contributing to the debates that have emerged from the existing literature, this research provides insights to reflect upon the legitimacy or illegitimacy of the selective interpretation of dissonant heritage and the power redistribution within the heritage system. Although postcolonialism criticises the clear-cut dichotomy of developed and developing nations, we still see such a dichotomy in reality, which is manifested by the World Heritage nomination process and the ICOMOS-influenced AHD of Kulangsu. The long-lasting call for greater inclusion of the Global South in the international heritage system has yet to be answered. Moreover, this international influence, together with other factors, such as the geopolitical situation and political agenda of China, contributes largely to the authorities’ selective interpretation of Kulangsu as a dissonant heritage, demonstrating that selective interpretation is a complicated process with intricate rationale.

Turning to the concept of AHD, it should not be seen purely as a static discourse but also as a dynamic process that reinforces the ideologies of the authorities while marginalising the masses. Selective interpretation of heritage value is de facto one of the processes within AHD that results in one dominant discourse and silences other discourses. In other words, selective interpretation is the very action that is supported by these uneven power relations between the authorities and the tourists and causes the tourists’ independent thinking and lay discourses to be downplayed by the authorities. Therefore, the reflection on the legitimacy of selective interpretation should not be confined to questioning whether controversial history is good or bad. Instead, how different actors interpret such controversial history and their motives for doing so are more important.

The major limitation of this research is that most of the data collection had to be conducted online due to travel restrictions during the COVID-19 pandemic. The problems are as follows: first, people without access to the internet, such as illiterate and elderly individuals, are not fully captured by the data. Second, although we tried to select tourists who had just travelled to Kulangsu or were in Kulangsu during the duration of this research, some of the respondents had travelled to Kulangsu a long time ago. Their memories and feelings about the trip may be somewhat faded. In the future, if more data could be collected in the field while tourists are visiting, they may reveal more about the research questions and complete this research. Another direction for future research is comparative research between different colonial heritage sites in China and between colonial heritage sites in China and those in other postcolonial countries. Since Kulangsu is considered to be a ‘moderate’ rather than a ‘radical’ concession, which is where more violence occurred, and since China is one of the few postcolonial countries that have been greatly revitalised, comparisons between ‘moderate’ and ‘radical’ concessions and comparisons between well-developed and less-developed postcolonial nations may be an interesting way to examine contestations and negotiations of heritage discourses. Despite these shortcomings, we believe that our research results reach beyond the study of colonial heritage in postcolonial countries and are inspirational for studying all types of dissonant heritage sites that have gone through constant reinterpretation and served nation-building and tourism development.

## Data Availability

Not applicable.

## Abbreviations

AHD: Authorised heritage discourse
THAD: Architectural Design & Research Institute of Tsinghua University Co. Ltd
UNESCO: United Nations Educational, Scientific and Cultural Organisation
ICOMOS: International Council on Monuments and Sites
OUV: Outstanding Universal Value

## Footnotes

1 Xiamen Urban Planning and Design Institute. 2020. Conservation Plan of Kulangsu Historical and Cultural District. Acquired from the interviewees.

2 In English, Kulangsu was a ‘concession’ as other concessions in China during that time. The Chinese translation is different, however. Kulangsu was a *gonggong dijie*, while other concessions were *gonggong zujie*. The difference is that the Chinese government remained sovereign in Kulangsu and could interfere with public affairs there, but had no such power in other concessions.

3 UNESCO website: The Selection of Criteria. https://whc.unesco.org/en/criteria/. Accessed 10 November 2021.

4 The Administrative Committee of Xiamen Kulangsu Scenic and Historic Area. 2016. Executive Summary of the Nomination of Kulangsu: A Historic International Settlement. https://whc.unesco.org/en/documents/155718. Accessed 15 August 2021.

5 ICOMOS. 2017. Evaluation of Nominations of Cultural and Mixed Properties. https://whc.unesco.org/en/documents/157742. Accessed 15 August 2021.

6 Same source as Note 5.

7 UNESCO website of Kulangsu. https://whc.unesco.org/en/list/1541.Accessed 15 August 2021.

8 ICOMOS on Shared Built Heritage. http://sbh.icomos.org/. Accessed 3 November 2021.
